# Multiple Roles and Women's Mental Health in Canada

**DOI:** 10.1186/1472-6874-4-S1-S3

**Published:** 2004-08-25

**Authors:** Heather Maclean, Keva Glynn, Donna Ansara

**Affiliations:** 1 Centre for Research in Women's Health, University of Toronto and Sunnybrook Women's Hospital, 790 Bay St., 7th Floor, Toronto, ON, Canada; 2 Centre for Research in Women's Health, University of Toronto and Sunnybrook Women's Hospital, 790 Bay St., 7th Floor, Toronto, ON, Canada; 3 Centre for Research in Women's Health, University of Toronto and Sunnybrook Women's Hospital, 790 Bay St., 7th Floor, Toronto, ON, Canada

## Abstract

**Health Issue:**

Research on the relationship between women's social roles and mental health has been equivocal. Although a greater number of roles often protect mental health, certain combinations can lead to strain. Our study explored the moderating affects of different role combinations on women's mental health by examining associations with socioeconomic status and differences in women's distress (depressive symptoms, personal stress (role strain) and chronic stress (role strain plus environmental stressors).

**Key Findings:**

Women with children, whether single or partnered, had a higher risk of personal stress. Distress, stress and chronic stress levels of mothers, regardless of employment, or marital status, are staggeringly high. Single, unemployed mothers were significantly more likely than all other groups to experience financial stress and food insecurity. For partnered mothers, rates of personal stress and chronic stress were significantly lower among unemployed partnered mothers. Married and partnered mothers reported better mental health than their single counterparts. Lone, unemployed mothers were twice as likely to report a high level of distress compared with other groups. Lone mothers, regardless of employment status, were more likely to report high personal and chronic stress.

**Data Gaps and Recommendations:**

National health surveys need to collect more data on the characteristics of women's work environment and their care giving responsibilities. Questions on household composition should include inter-generational households, same sex couples and multifamily arrangements. Data disaggregation by ethno-racial background would be helpful. Data should be collected on perceived quality of domestic and partnership roles and division of labours.

## Background

The effect of multiple roles on women's psychological well-being remains controversial. [1-3] Although it is well recognized that women's social roles affect their mental health, it is unclear whether the effects are beneficial or detrimental. [4] Traditionally, this research has been conducted within two competing hypotheses: role strain theory proposes that because each person has limited time and energy, women with multiple roles often experience "role conflict," which results in harmful effects on their mental and physical health. [5,6] The opposing theory suggests that each additional role brings benefits, including increased social contacts and self-esteem, which contribute to better health and greater psychological well-being. [7]

More recent research indicates that involvement in each role has both harmful and beneficial effects, and the balance between these varies depending on the characteristics of the role, the specific combination of roles, and the socio-economic context of women's lives. [8] Socio-economic status creates different experiences and exposures in daily life and these, in turn, have consequences for women's psychological health. [9-11] Of particular importance is the finding that as women's education and income levels rise, there is a decline in distress levels. [11] In terms of combinations of roles, there is mixed evidence as to the effects of various combinations on women's psychological well-being. Although more roles often protect mental health, certain combinations can also lead to strain. [12,13]

### Employment

The majority of previous studies have shown that women employed outside the home tend to experience better mental health outcomes than those who are not employed outside the home, [2,14,15] depending upon the specific characteristics of the job and the level of social support at work. Other research has found no significant difference in the mental health outcomes between employed and unemployed women. [7,11-16] Repetti et al. compared the distress levels of employed and unemployed single and married women, and found that employment was associated with improved mental health for both groups. [17] A more recent study, which used a large, national sample, found that mental well-being was positively related to the mother's employment and negatively related to her total number of children. [18] With respect to the quality of the work environment, a recent Canadian study [19] found that women who worked long hours had increased odds of subsequently experiencing depression when compared with women who worked standard hours. In a related study, shift work was also shown to be associated with increases in psychological distress among women. [20] Wilkins and Beaudet [21] studied the impact of work stress on health and found that job insecurity was associated with migraine headaches among women. Further, they found that low co-worker support was related to both work injury and psychological distress.

### Partnership and Marriage

Marriage is also thought to be protective of health for women, mainly because it offers social support and financial resources. [22-24] Lahelma et al. looked at different family and parental role combinations in Britain and Finland, and found that women living in two-parent families with children reported better health than women living in other family types or on their own. [25] In particular, lone mothers showed worse health overall relative to other women. These results are reinforced by a Canadian study [26] that found that lone mothers had lower rates of self-reported health and happiness and higher levels of distress than mothers in two-parent families.

Research has also shown that marriage and employment may moderate one another. A recent study found that a marriage in which the wife had no earnings was more than twice as likely to dissolve, as was a marriage in which the woman earned between US$1,000 and US$18,000. [27] In terms of unpaid work, Bird analyzed data from a national U.S. database to evaluate the relation between the division of household labour by married couples, and depression. [28] The results revealed that it was the inequity of the division of labour that was the important predictor of depressive symptoms rather than the absolute number of hours worked.

### Parenthood

In contrast to work and partner roles, the health effects of being a parent are less clear. However, there is strong evidence that parenthood tends to be a particularly stressful experience for working-class mothers. [4,29] Numerous studies have shown that being a lone mother is particularly disadvantageous, both economically and in terms of mental health. [30,31] Evidence of high levels of distress among lone mothers is reinforced by findings from Hall's study on lone mothers' experiences in the workforce: [32] lower economic status and lower-quality work led to substantial levels of distress among lone mothers relative to their partnered counterparts.

### Multiple Roles, Socio-economic Status and Psychological Health

Studies considering women's distress levels according to role combinations and socio-economic status have had mixed results. Although there is strong evidence to support a socio-economic gradient in women's distress levels, the extent to which differences in women's roles contribute to this gradient remains unclear. [11]

### Multiple Roles and Immigrant Women

The need for economic sufficiency has had a substantial impact on the roles of immigrant women in Canada. [33-35] Studies of a number of different immigrant groups, including Sri Lankan Tamil, South Asian and Italian women, found that after migration to Canada immigrant women were more likely to participate in the workforce than if they had stayed in their home country. [34-36] Many immigrant women try to maintain a balance between their traditional role expectations and Canadian lifestyle and expectations. [36] As a result, women continue to perform the majority of domestic labour while also maintaining employment outside the home. The competing demands of family and work are exacerbated by the larger challenge of balancing traditional and Canadian values. [33-35] The changing roles of immigrant women have been shown to affect their mental health, and in particular to put them at greater risk of depression. [37]

### The Current Study

Our study examined NPHS data to further explore the moderating effects of different role combinations on women's mental health and to situate this analysis in a social context. To accomplish this we examined (1) the association between different role combinations and socio-economic status, and (2) the differences in women's stress, distress and chronic stress levels according to the various combinations of roles.

## Methods

### Data

Cross-sectional data from the NPHS (1994–1995 and 1998–1999) were analyzed. The data for this report were obtained from individuals aged 15 to 64 and were weighted to reflect the population of Canadian women at the time of each survey. In the NPHS (1994–1995) cycle there were 7,364 women aged 15 to 64 years of age, of whom 60.82% had at least one dependent child. In the NPHS (1998–1999) cycle there were 6,087 women aged 15 to 64 years of age, of whom 61.29% were mothers.

### Measures

#### Social Roles

In order to contrast the mental health effects of parenthood, this analysis includes both women with and women without children, as appropriate. With respect to marital status, we contrasted those who were single (i.e. never married, widowed, divorced or separated) with those who were partnered (i.e. legally married or in a common-law relationship). Finally, those engaging in paid work versus those engaging in unpaid work were compared. Thus the following role profiles were examined:

1. single parent, not employed

2. single parent, employed

3. partnered with children, not employed

4. partnered with children, employed

5. women without children, partnered and single.

Respondents who were retired at the time the survey was conducted were excluded from the report.

#### Health Outcomes

Distress was examined using a six-item scale that assessed feelings of sadness, of anxiety, of hopelessness, of worthlessness, and the feeling that everything had been an effort within the previous month. The scale, based on work conducted by Kessler and Mroczek of the University of Michigan, is derived from the Composite International Diagnostic Interview. Scores on the distress scale range from 0 (no distress) to 24 (highly distressed). Because there is no agreed-upon definition of high distress, we identified those scoring 5 or more (representing the 75th percentile for women) as experiencing high distress, and those scoring between 0 and 4 as having no or low distress. [42]

Stress comprised two measures, personal stress and general chronic stress. Personal stress measures whether respondents experienced any of five different role stressors, consisting of trying to take on too much at once, feeling pressure to be like other people, feeling that others expect too much, feeling that work around the home is not appreciated, and feeling that others are too critical. General chronic stress includes the same five items as those in the personal stress index in addition to several other stressors: experiencing financial stress; having friends that are a bad influence; having a desire to move but being unable to do so; living in a neighbourhood or community that is too noisy or polluted; having an ill parent, partner or child; and having a family member with an alcohol or drug problem. For both stress measures, those scoring at or above the 75th percentile were identified as having a high level of stress (personal stress score of 2 or more, general chronic stress score of 3 or more).

### Statistical Analysis

The variability of the data was determined using the approximate variance tables provided by Statistics Canada. Estimates that have a high coefficient of variation (CV) (between 16.6% and 33.3%) should be interpreted with caution. In accordance with Statistics Canada's guidelines, estimates that were based on a sample of fewer than 30 were suppressed because of the unreliability of the estimate. Statistical tests were conducted using the weighted proportions and the CV estimates obtained from the approximate variance tables. Estimates of the proportion of women with distress, personal stress and general chronic stress by multiple role profiles were presented by age (15–29, 30–39, 40–64).

## Results

**Figure 1:**
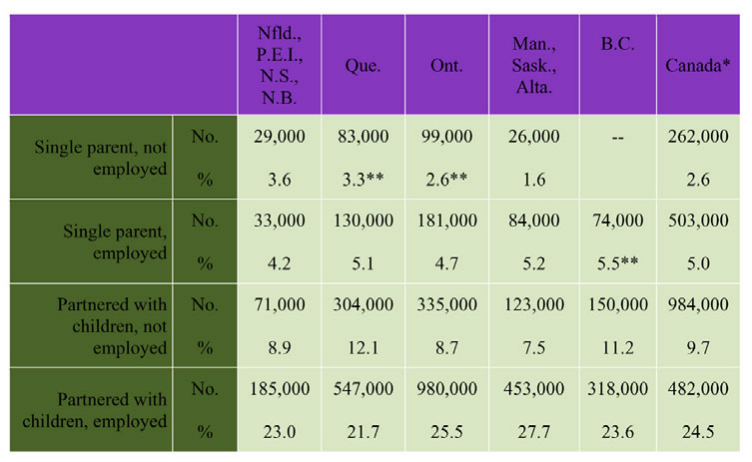
Social Role Profiles of Mothers, Canada and the Provinces, 1998–1999 --  Unable to report data (number surveyed is less than 30) *  The denominator includes all women in Canada between the ages of 15 and 64. ** Coefficient of variation is high (16.6% to 33.3%)

**Figure 2:**
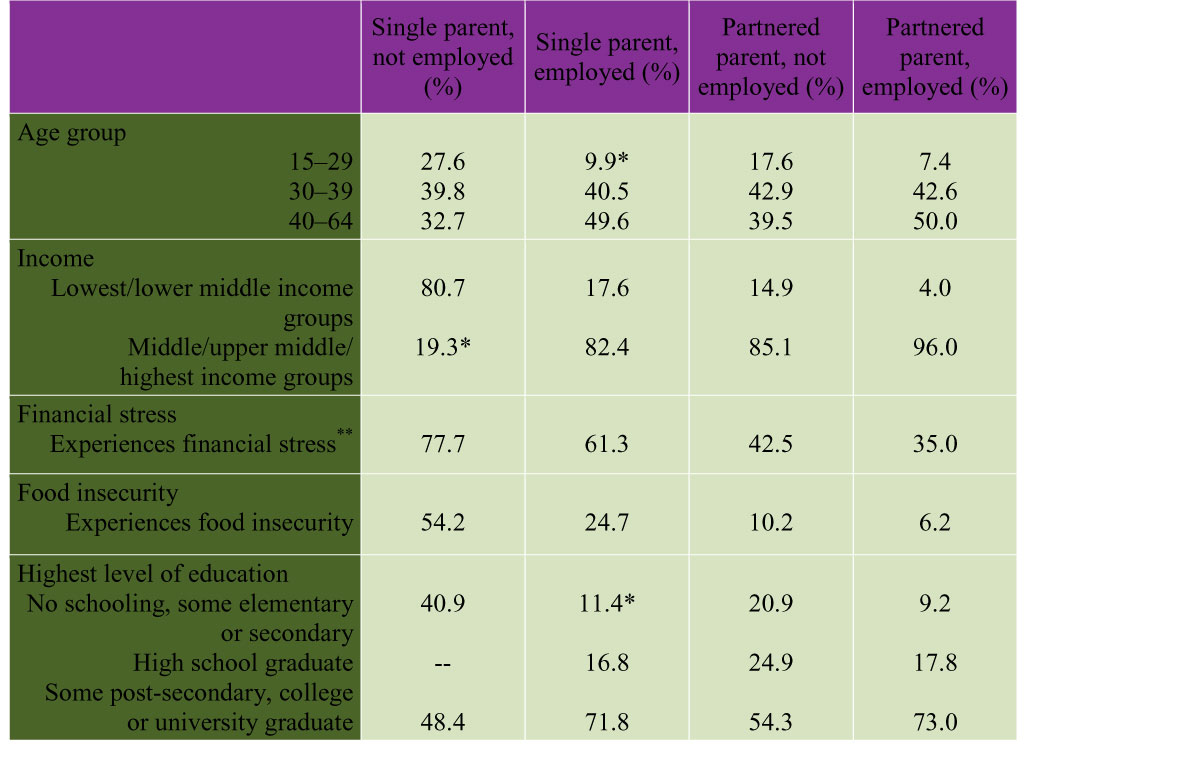
Demographic Characteristics of Women in Role Profiles, Canada, 1998–1999 -- Unable to report data (number surveyed is less than 30) * Coefficient of variation is high (16.6% to 33.3%)

**Figure 3:**
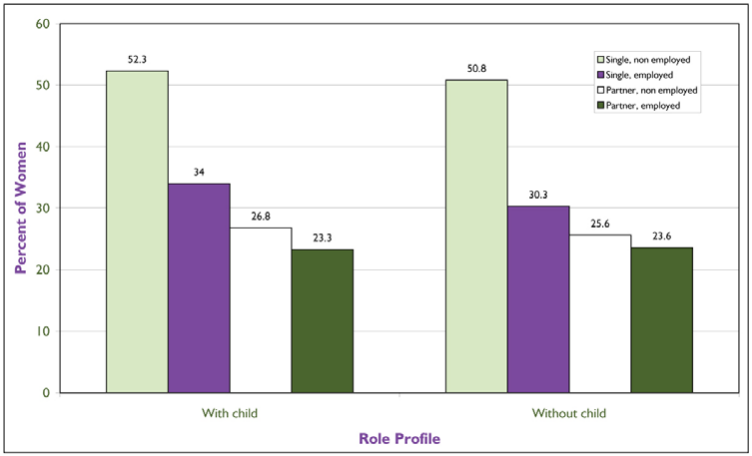
Percent reporting high psychological

**Figure 4:**
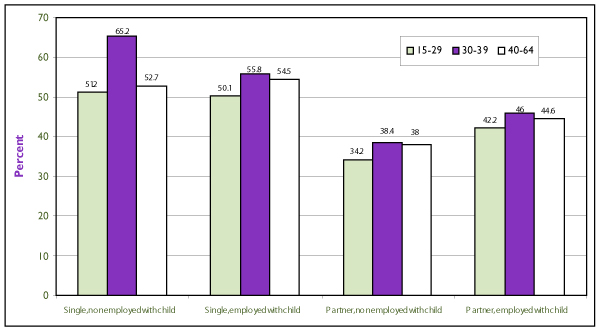
Proportion of Women With High Personal Stress by Multiple Roles and Age, Canada, NPHS, 1994–1995

**Figure 5:**
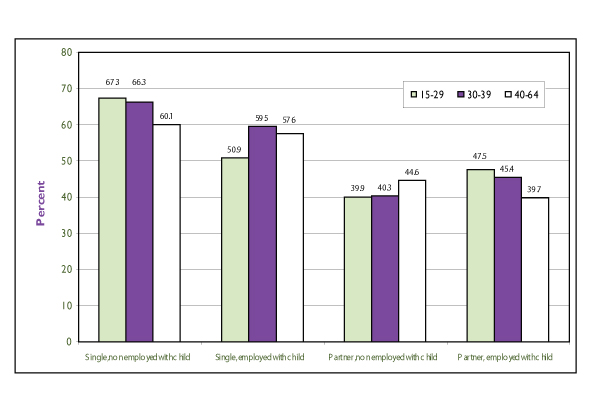
Percent Reporting High Chronic Stress by Multiple Roles and Age, Canada, NPHS, 1994–1995

The proportions presented in Figure 1 reflect the number of mothers aged 15 to 64 who occupied the various role profiles out of the total number of women aged 15 to 64 in Canada and the regions. Figure 2 displays the socio-demographic characteristics of mothers within the various roles, and Figures 3, 4 and 5 depict the proportion of mothers within the roles who reported a high level of distress, personal stress, and general chronic stress. The denominators for these proportions are based on the number of women (weighted) who occupied the specific roles.

### Geographic Variation*

The Atlantic provinces and Quebec stood out among Canadian provinces and regions as having the highest percentage of unemployed mothers (Figure 1); among single, unemployed mothers the proportions were 3.6% and 3.3% respectively. Quebec also reported the highest percentage of partnered, unemployed mothers (12.1%). The Prairie provinces had the highest proportion of employed, partnered mothers (27.7%). Finally, although the concentration of single working mothers was comparable across all regions, British Columbia had a slightly higher percentage (5.5%).

### Multiple Roles and Socio-demographic Characteristics

As expected from our review of the literature, lone mothers were significantly more likely than partnered mothers to be poor and to experience financial stress and food insecurity, irrespective of their employment status. With respect to employment, whether partnered or single, employed mothers were significantly more likely to be older and to have higher levels of education than non-employed mothers.

Single, unemployed mothers were significantly more likely than all other groups to fall into the youngest age group (27.6%) (Figure 2). Also, partnered mothers who were not employed (17.6%) were more likely than employed single or partnered mothers to fall into the younger age group. With respect to socio-economic status, an overwhelming majority of single mothers who were not employed fell into the low-income bracket (80.7%), as compared with only 4% of partnered, employed mothers. Single, employed mothers and partnered, unemployed mothers were equally likely to report low incomes (17.6% and 14.9% respectively). In terms of financial stress and food insecurity, all groups were significantly different from one another. Single parents who were not employed were more likely than all other groups to report financial stress (77.7%) and food insecurity (54.2%), whereas partnered women who were employed were least likely (35.0%, 6.2%) to do so. Finally, unemployed, single parents were more likely than all other groups to have less than a high school education (40.9%). Partnered mothers who were not employed were more likely than employed single and partnered mothers to have less than a high school education (20.9% versus 11.4% and 9.2% respectively).

### Multiple Roles and Stress

Lone mothers who were not employed were more than twice as likely as all other groups to report a high level of distress (Figure 3). Of older (40 to 64 years) lone mothers who were not employed, 60.9% reported high distress levels, as compared with only 20% of partnered mothers in the same age cohort who were not employed. No substantial differences were found between women in the remaining three groups.

In all age groups, lone mothers (both employed and unemployed) were most likely to report feelings of high personal stress and feeling overloaded, whereas partnered mothers in all age groups who were not working for pay were least likely to do so (Figure 4). Mothers who were partnered and working fell these groups.

With respect to chronic stress, lone mothers, irrespective of employment status, were more likely than partnered mothers to report high levels of chronic stress (Figure 5). Again, in all age categories, partnered mothers who were not employed were the least likely of the four groups to report chronic stress.

**Figure 6:**
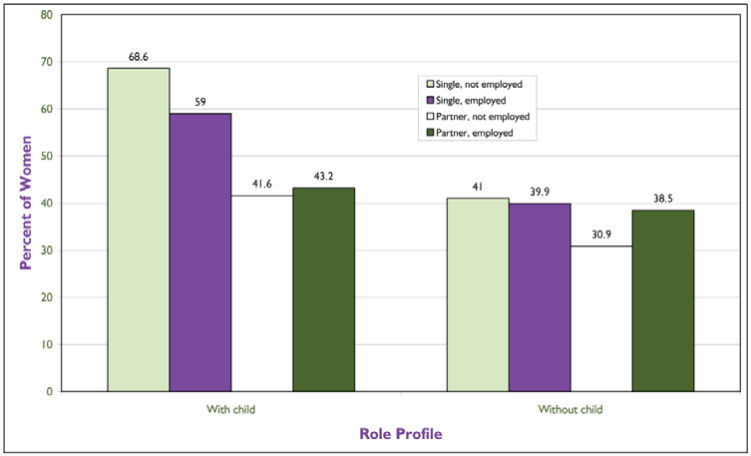
Percent Reporting High Personal Stress for Women in Role Profiles, Canada, NPHS, 1994–1995

**Figure 7:**
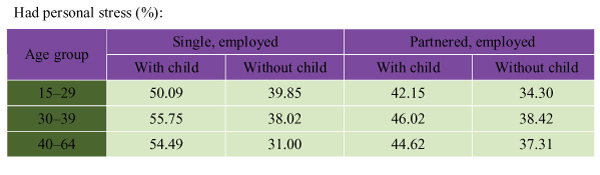
**Percentage of Women Reporting High Personal Stress by Age Group, With and Without Children. **Source: Statistics Canada, National Population Health Survey, 1994–1995

**Figure 8:**
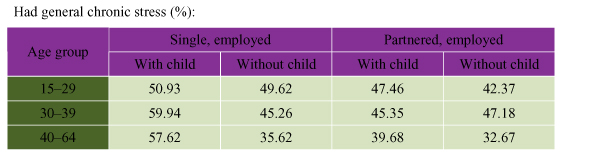
**Percentage of Women Reporting High Chronic Stress by Age Group, With and Without Children. **Source: Statistics Canada, National Population Health Survey, 1994–1995.

Finally, women with children, whether single or partnered, had a higher risk of personal stress when compared with those without children (Figure 6). The effect was more pronounced in our comparison of single women with and without children than that of partnered women with and without children. We also observed these results when stratifying for age group, although samples were small in some categories (Figures 7 and 8). With respect to high psychological distress, we found no differences between women with and without children.

## Discussion

### Geographic Variation

The regional differences in the types of roles women tend to occupy were most pronounced in the Atlantic provinces, Quebec and British Columbia. The relatively high percentage of non-employed, single parents in the Atlantic provinces reflects the high unemployment rates for women and the weak economy of the eastern provinces, particularly of Newfoundland and Labrador and Prince Edward Island. [38] The relatively high numbers of single, employed mothers in British Columbia and single (employed and unemployed) mothers in Quebec appear incongruous in relation to the relative wealth of these two provinces, but they may be due, in part, to the cultural differences across regions. Further investigation into the regional differences is warranted, as they appear to be only partially explained by provincial disparities in wealth.

### Multiple Roles and Socio-demographic Characteristics

Consistent with prior research, [24,25] mothers who were married or partnered tended to be better off financially than single mothers, regardless of employment status. Further, single, unemployed mothers were significantly more likely than all other groups to experience financial stress and food insecurity; as expected, they also reported lower levels of education and were more likely to be in the younger age cohort. In contrast, partnered, employed mothers were significantly less likely than single, employed mothers to experience financial stress or food insecurity. Our results with respect to education and mothers' employment were consistent with national trends indicating an increase in employment as education levels rise. [39] Mothers who participated in the paid workforce, whether single or partnered, were significantly more likely to have a post-secondary degree than mothers who were not employed outside the home. Trends in terms of women's age and employment status were also consistent with national statistics, in that employed women (both single and partnered) were significantly older than their unemployed counterparts. [39]

### Multiple Roles and Distress/Stress

Our results in this area were mixed. Our finding of no difference in distress between women with and without children reflects the fact that distress measures depressive symptoms, and the mere presence (or absence) of children in the household has not been shown in the literature to be a strong predictor of depression in women. Our comparison of single employed and non-employed mothers supports prior research indicating lower rates of distress for employed, lone mothers as compared with single, non-employed mothers. This may be due to both increased financial stability and the proven mental health benefits of tempering stress in the domestic sphere with work pursuits. [2,14,15]

With respect to partnered mothers, our results were distinct. For partnered mothers, employment did not have a significant effect on distress or chronic stress levels. However, rates of personal stress – possibly the closest measure representing role overload – were significantly lower among partnered mothers who were unemployed than among those who were employed. As such, this finding could be interpreted as further evidence to support role strain theory. In this case, our mixed results may also indicate the need for the use of standardized variables across different studies to measure role overload with respect to multiple roles.

As demonstrated elsewhere, [24,25] married and partnered mothers reported better mental health than their single counterparts. As expected, our study demonstrated significantly higher rates of personal stress and chronic stress for single employed and unemployed mothers in comparison with partnered mothers. Further, unemployed single mothers reported significantly higher rates of distress than unemployed, partnered mothers.

### Limitations of the Analysis

Despite extensive research on the relation between the quantity and quality of social roles and health, it remains unclear to what extent women's roles affect their physical and mental health. The lack of clarity is due, in part, to inconsistent use in the literature of concepts and definitions such as multiple roles, social support and employment.

In our study, we encountered a number of limitations with respect to the NPHS data. First, because the study was cross-sectional, we were unable to determine causal chains for the outcomes measured. Second, the sampling strategy used in the study focused on household dwellings and excluded specific populations. Two of these populations, Native women living on reserves and homeless women, likely include a high proportion of lone mothers. However, these excluded populations represent a small proportion of Canada's population, so it is possible that the number of women excluded was small. Third, the NPHS does not include data on the conditions within the home, the domestic responsibilities of women, or the household division of labour and caregiving. This is a substantial gap in the data, as numerous studies have shown that parenting young children places women at additional risk of psychological disturbance. [40,41] The NPHS imposed similar restrictions with respect to determining the quality of role experiences outside the home, most notably employment. Of particular concern for our analysis was the inability to distinguish between voluntary and involuntary unemployment using NPHS data. As a result of this limitation, our findings may be confounded and should be interpreted with caution. This problem is not believed to be major since employment status (employed, unemployed and "not in the labour force") does not appear to vary greatly between partnered and single mothers (26).

## Recommendations

Despite the limitations, it is clear that the distress, stress and chronic stress levels of mothers, regardless of employment or marital status, are staggeringly high. The evidence was most striking for single, non-employed mothers, whose levels were markedly higher than those of all other groups. Employment appeared to play a protective role for women with respect to distress but exacerbated personal stress levels among partnered women. Clearly, further research is needed in this area to better understand the effects of different roles and role combinations on women's personal stress levels. We propose the following directions for future data collection and research.

### Data Collection

• More detailed information needs to be collected on the characteristics of women's work environments, particularly scheduling (part-time versus full-time work, the flexibility of hours, on call and shift work etc.).

• More detailed information needs to be collected on women's responsibilities with respect to the care-giving provided to both the very young and the very old.

• Future national surveys should extend questions related to household composition to include intergenerational households, households headed by same-sex couples and multi-family arrangements.

• More information is needed on the quality of women's domestic roles, more specifically on the number of years of marriage/partnership, women's subjective assessment of their partnership, and the division of labour.

• Disaggregated information on women's ethno-racial backgrounds would be helpful for future research and data on various subgroups of women (e.g. lesbian, First Nations women).

### Policy Recommendations

• Labour force policies and policies to support family life need to be developed. Integral to these policies is the recognition of women's participation in the labour force and as unpaid caregivers in the home.

• Our findings indicate the importance of expanding the child-care and economic subsidies that are available to lone mothers. A comparative analysis of the impact of these different policies on the mental health of lone mothers would provide important information for Canadian policy analysts.

• Employment strategies specific to lone mothers should be developed. Given the substantial number of reports of high distress (and the smaller, but still present, differences in stress and chronic stress) among unemployed lone mothers, employment strategies that consider the special needs of lone mothers should be developed.

• Educational programs to enhance mental health professionals' understanding of the impact of multiple roles on women's mental health should be put in place.

## Note

* Because of insufficient sample sizes we were unable to disaggregate the data by province.
